# Data Diversity in Convolutional Neural Network Based Ensemble Model for Diabetic Retinopathy

**DOI:** 10.3390/biomimetics8020187

**Published:** 2023-04-30

**Authors:** Inamullah, Saima Hassan, Nabil A. Alrajeh, Emad A. Mohammed, Shafiullah Khan

**Affiliations:** 1 Institute of Computing, Kohat University of Science and Technology (KUST), Kohat City 24000, Pakistanskhan@kust.edu.pk (S.K.); 2 Biomedical Technology Department, College of Applied Medical Sciences, King Saud University, P.O. Box 10219, Riyadh 1433, Saudi Arabia; 3 Department of Engineering, Faculty of Science, Thompson Rivers University, 805 TRU Way, Kamloops, BC V2C 0C8, Canada

**Keywords:** diabetic retinopathy, ensemble models, machine learning, deep learning, convolution neural network

## Abstract

The medical and healthcare domains require automatic diagnosis systems (ADS) for the identification of health problems with technological advancements. Biomedical imaging is one of the techniques used in computer-aided diagnosis systems. Ophthalmologists examine fundus images (FI) to detect and classify stages of diabetic retinopathy (DR). DR is a chronic disease that appears in patients with long-term diabetes. Unattained patients can lead to severe conditions of DR, such as retinal eye detachments. Therefore, early detection and classification of DR are crucial to ward off advanced stages of DR and preserve the vision. Data diversity in an ensemble model refers to the use of multiple models trained on different subsets of data to improve the ensemble’s overall performance. In the context of an ensemble model based on a convolutional neural network (CNN) for diabetic retinopathy, this could involve training multiple CNNs on various subsets of retinal images, including images from different patients or those captured using distinct imaging techniques. By combining the predictions of these multiple models, the ensemble model can potentially make more accurate predictions than a single prediction. In this paper, an ensemble model (EM) of three CNN models is proposed for limited and imbalanced DR data using data diversity. Detecting the Class 1 stage of DR is important to control this fatal disease in time. CNN-based EM is incorporated to classify the five classes of DR while giving attention to the early stage, i.e., Class 1. Furthermore, data diversity is created by applying various augmentation and generation techniques with affine transformation. Compared to the single model and other existing work, the proposed EM has achieved better multi-class classification accuracy, precision, sensitivity, and specificity of 91.06%, 91.00%, 95.01%, and 98.38%, respectively.

## 1. Introduction

In the era of big data, data are considered precious. The advancement of two paradigms, big data and parallel processing, has revolutionized the fields of data science and artificial intelligence (AI). AI models have shown tremendous performance on different regression and classification problems. Visual phenomena, or computer vision (CV), is today’s hot topic to address the hidden patterns in images. The basic purpose of developing a mathematical or statistical model is to automatically acquire, annotate, and understand the images. Accuracy plays a vital role in the biological and medical fields. Medical image analysis (MIA) retrieves valuable information from different medical imaging modalities to detect fatal diseases in time. The common medical imaging modalities are X-ray, magnetic resonance imaging (MRI), optical coherence tomography (OCT), computed tomography (CT), positron emission tomography (PET), CT-Scan, histopathology, mammography, endoscopy, fundus images (FI), etc. [1]. 

Clinicians, radiologists, and healthcare personnel use these gold-standard techniques to diagnose various diseases. However, these techniques are prone to error and are time-consuming. In addition, the data are annotated by medical domain experts who anticipate the outcomes manually. In cases of the non-availability of domain experts or the presence of unskilled healthcare personnel, a wrong interpretation of the image may cause severe problems for the patient. Therefore, it is essential to improve and develop an efficient model to assist radiologists, clinicians, and other medical staff in analyzing and diagnosing different mortal diseases [2,3]. Decreasing insulin production can result in a common medical condition termed diabetes mellitus (DM). DM may be type 1 or 2. The pervasiveness of type-2 diabetes in diabetic patients has been shown to be 90%. The prevalence of type 2 can cause other diabetic diseases, such as DR. DR is a chronic disease, and if left unattended, it may lead to severe eye conditions such as retinal detachment or blindness. It is one of the leading causes of blindness and is generally found in patients aged 20 to 65. The ophthalmologist has classified DR into five main classes, including Class 0 (no DR), Class 1 (mild), Class 2 (moderate), Class 3 (severe), and Class 4 (proliferative DR) [4,5,6]. The formation of various forms of lesions on the retina of the eyes recognizes DR. The different types of lesions are microaneurysms (MA), hemorrhages (HM), soft exudates, and hard exudates. MA is a small red round dot that appears as a lesion size less than 125 µm, while HM is a large red dot of size 125+ µm. Similarly, neovascularization is another lesion that is severe and can lead to retinal detachment. Class 0 means a normal eye or a patient with no DR. Class 1 patients have a single MA lesion, while Class 2 patients have more than one MA lesion. 

**Figure 1:**
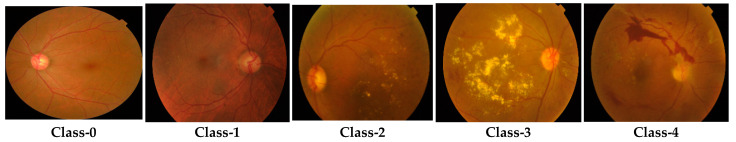
Five stages of DR.

Similarly, in Class 3, patients have more than 20 intra-retinal HM lesions in every four quadrants. In the last and final stage, “Class 4” has a sign of a neovascularization lesion and has a proliferative stage in which a person can become completely blind [7]. Additionally, Figure 1 presents the pictorial view of five classes of DR. The initial stages of DR are very important to prevent and control the disease on time and diagnose it as early as possible. DR is observed through FI. However, an expert is required to recognize and detect the lesions and stages of FI. Computer vision (CV) has been widely used for the past two decades to interpret and diagnose various stages of FI [8]. Nowadays, CV researchers follow two techniques, such as hand-engineering and end-to-end learning. Both techniques have their advantages and disadvantages. End-to-end learning is a new technique that does not require handcraft engineering, and the model automatically learns the features and classifies itself. In end-to-end learning, CNN is widely used for retrieval and visual inspection. CNNs have the power to extract valuable information from images and interpret it into the required outcomes. In medical imaging, CNNs have held a unique position for a decade [9].

Over time, researchers have introduced new models, such as the ensemble model (EM). In EM, the output obtained from the individual models is aggregated into a new model [10,11]. The core idea behind EM is to combine multiple models so that other models compensate for the errors of an individual model. CNN-based EM is a new approach that has been used to solve different classification and regression problems for the past few years and has shown great success [12]. In developing countries, while working in the area of MIA, data paucity or imbalanced data is often encountered due to ethical requirements and privacy constraints. Data paucity refers to situations where there is a lack of data, while imbalanced data refers to situations where there are uneven classes or unequal outcomes for each class. Scarcity and imbalanced data can greatly affect the accuracy and efficiency of the automatic diagnostic model [13]. 

To deal with these shortcomings, an EM based on the CNN model is incorporated into this work. The potency of the EM model can deliberately improve the performance measures of the above-mentioned dataset. DR (APTOS-2019) is a publicly available Kaggle dataset released in the third quarter of 2019 [14]. It is used in this research work as a benchmark to construct an automatic diagnostic model. The total of train and test labels comprises 3662 images. They are further classified into five classes, namely 0, 1, 2, 3 and 4. In this research, we have implemented EM based on the three-CNN models on DR-fundus images to classify the five classes with limited and imbalanced medical datasets. In addition, data diversity in the proposed model is created through different data augmentation techniques, such as position and color augmentation. Each model in the ensemble uses different data, which introduces data diversity in the proposed model. The rest of the paper is organized as follows: The Section 2 discusses recent existing studies, followed by a Section 3, describing the methods used in this research. The CNN-based EM is explained in the Section 4. Section 5 discusses the obtained results, while the conclusion, limitations, and future direction are highlighted in the final section.

## 2. Related Works

**Figure 2:**
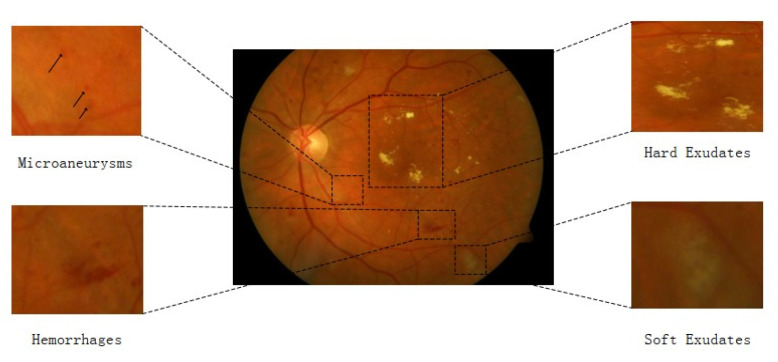
Annotated results of an image with DR [4].

DR is mostly diagnosed manually by inspecting the retinal images. The process is time-consuming and challenging since some lesions in the retinal image are tiny or subtle, such as microaneurysms illustrated in Figure 2. Many automatic or semi-automatic approaches used in CV and machine learning algorithms have been applied to increase the efficiency and accuracy of DR classifications. Before the advent of deep learning (DL) algorithms, feature extraction was a mandatory step in image classification, as in conventional CV methods. These features contain some specific and very important information about the image. Many feature extraction algorithms were proposed in the 1990s, such as SIFT [15] and SURF [16], which have been widely applied for object recognition and MIA [2]. The manually extracted features are excessively defined, incomplete, or require a long time and skill to construct and test when using traditional CV methods for DR lesion identification or classification. Instead of manual feature extraction for DR screening, many researchers are now focusing on end-to-end DL models that automatically learn all the needed features. 

Here, we review previous studies regarding DR based on the newly trended CNN model and categorize the DR into binary and multi-classifications. K. Xu et al. [17] proposed a CNN model to detect the DR on time. The models were validated on the Kaggle dataset [14]. The authors used one thousand FI. Before training, the model augmented the images with different affine techniques to increase the number of images. The CNN model was utilized and classified the DR disease into binary classifications. The DR was categorized into binary classes with a significant result of 94.5% accuracy. In a recent study, the binary classification of DR disease was performed using CNN [18]. The ResNet34 model was utilized to classify the FI into DR or no-DR images. They used 35,000 images from the DR Kaggle dataset. Different pre-processing techniques are utilized, such as the Gaussian filter, weighted addition, and normalization, to robustly improve the performance of images. After image pre-processing, the Resent34 model was trained and validated to conclude the result with 85% accuracy and 86% sensitivity.

Jiang et al. [19] proposed EM through Adaboost algorithms to diagnose the DR binary classification automatically. The authors collected 30,244 FI with the collaboration of “The Beijing Tongren Eye Center” to train the model. Three pre-trained (PT) models (Inception V3, Resnet152, and Inception-Resnet-V2) were integrated into a single network goal to improve the results. This framework obtained 85.57% sensitivity, 88.21% accuracy, 90.85% specificity, and a 0.946 AUC. In [20], the proposed work of the custom CNN and PT model (VGG16) first identified the lesion and then classified it as referable or non-referable DR. The best result achieved was a value of 0.94% and 0.912% of sensitivity and AUC, respectively. Similarly, Harangi et al. [21] included the publicly available dataset [22] in their studies and categorized the DR into a multi-class classification. The authors utilized end-to-end DL and traditional ML to detect the disease in time with an accuracy of 90.07%. X. Li et al. [23] classified the dataset used in [22] as referable and non-referable images and categorized the public dataset [24] as five DR stages and three diabetics macular edema stages, using the ResNet50 and four attention modules. ResNet50′s features were used as inputs for the first two attention modules, selecting one disease feature. The first two attention modules have average pooling, maximum pooling, multiplication, concatenation, convolution (Conv), and fully connected (FC) layers, while the next two contain FC and multiplication layers. Data augmentation, standardization, and resizing were performed before feeding the images to CNN. For the dataset in [22], the study produced a sensitivity of 92%, an AUC of 96.3%, and an accuracy of 92.6%. In [25], the proposed method used end-to-end learning algorithms consisting of 10 Conv, eight max pooling, and three FC layers. Two general techniques, L2 regularization and dropout, were performed to diminish variance, outperforming the results. As a result, 95% specificity, 30% sensitivity, and 75% precision were attained successfully. In the same way, Qummar et al. [7] ensembled five PT networks, such as Resent50, inceptonV3, exception, Dense-121, and 169, and validated them on the Kaggle dataset. The accuracy, recall, specificity, precision, and F-1 scores obtained were 80.8%, 51.5%, 86.72%, 63.85%, and 53.74%, respectively. The authors [26] employed a hybrid of PT and CNN models on the dataset [14], which has intensified the existing model’s accuracy. The hybrid model attained an accuracy of 82.18%. 

Likewise, Mehboob et al. [6] suggested a model of three frameworks in their study on the dataset [14] and picked up an ensemble framework. The ensemble framework outperformed the results from the single models used in the framework and has classified the DR into multiple classes. The accuracy yielded from this model was 78.06 and 83.78% with or without affine techniques, respectively.

## 3. Background

This section briefly describes the methods used in this research.

### 3.1. Ensemble Model

EM aims to build a predictive model by integrating multiple models to improve prediction performance. Researchers from various disciplines, including statistics, economics, and computer science, have employed the EM technique in their research. EM is generally constructed in two steps: baseline model generation and model combination. EM consists of multiple classifiers called baseline models. Support vector machines (SVMs), random forests (RFs), and neural networks (NNs), as well as any other machine learning (ML) algorithm, can be used to generate these baseline models. EM may be homogeneous or heterogeneous [27]. In a homogeneous EM model, identical baseline models are utilized (such as a cluster of several SVMs, NNs, or CNNs). Likewise, in heterogeneous EM, different baseline models are generated while keeping the data the same. Then, these diverse models (homogeneous or heterogeneous) are combined for Ensembling [28]. 

Nothing can be gained with an ensemble of identical models. Thus, it was needed to have diversity in the individual models while blending. In an attempt to alleviate the accuracy and stability of the EM, a diverse set of individual models is the best choice for aggregation. The construction of EM is based on various methods, but the most prominent methods are bagging and boosting [29]. The bagging method constructs an ensemble by generating multiple copies from the training examples. After multiple copies are created, they are combined with the same or different algorithms. Due to this phenomenon, bagging is also termed bootstrap aggregating. The EM is combined using different techniques; however, majority voting is ideal for classification problems. Bagging also helps in classification problems to reduce the variance (overfitting) [30]. Boosting is a meta-learning algorithm that combines a weak classifier to create a strong classifier. It follows an iterative process to tackle the errors and construct a new model. It incrementally constructs an ensemble by iteratively training a new model to emphasize misclassified training samples from previous models. Boosting helps reduce bias (underfitting) in classification problems [31]. 

Diversity plays a key role in EM and can be created using three main techniques: data, parameter, and structural diversity. Data diversity is created by training the original data to manipulate the input characters in disjoint or repeated ways. Parameter diversity is achieved by changing the hyperparameter to obtain a variety of baseline models. The parameter diversity-based baseline models work with varying degrees of fit, introducing diversity. Similarly, structure diversity is attained by changing the structure of the baseline models, or it can be created by combining different models that can serve as baseline models (as in heterogeneous ensembles) [12]. The focus of this paper is on data diversity, and its proximity and methodology are identical to bagging techniques for generating and aggregating models.

### 3.2. Convolutional Neural Network (CNN)

CNN, also known as ConvNet, is a DL model almost universally used in different CV tasks. CNN is inspired by the mammal’s visual cortex framework. The inception of modern CNN dates back to 1998, when CNN architecture was introduced to classify handwritten digits [32]. With advancements in the field, several types of CNN have been developed and significantly contribute to the performance of automatic image identification. These models include architectures such as AlexNet, GoogleNet, VGGNet, ResNet, etc. In addition to these networks, one can quickly build an efficient model from scratch with its structural design using the modern programming platforms and libraries available today. CNN typically has three layers: convolution, pooling, and an FC layer.

**Figure 3:**
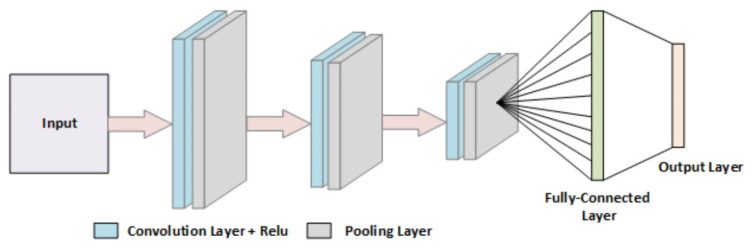
Basic CNN Architecture.

Figure 3 shows the basic architecture of a CNN model. The first two layers perform feature extraction in a hierarchical form (from low- to high-level patterns). CNN has an input layer that takes normalized image data of the same size. After that, images are passed to a convolutional layer, the main building block of CNN. This layer automatically learns enormous filters parallel to a dataset for a specific problem, such as classification. The resultant features are highly specific and can be observed anywhere on an input image. After the convolutional layer, the data goes through another building block of CNN known as the pooling layer. Its purpose is to gradually decrease the spatial size of the image to minimize the quantity of computation and parameters contained in a network. It works on every feature map individually. In contrast, the FC layer maps the extracted features into the final output, such as classification.

## 4. Proposed Methodology

**Figure 4:**
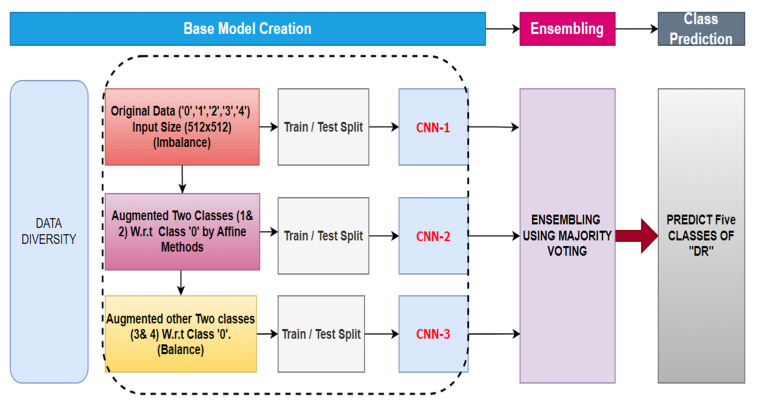
Workflow of the proposed model.

The proposed methodology of this research work is depicted in Figure 4. The data sets utilized in this investigation are presented in Section 4.1 and Section 4.2, respectively. The baseline model creation and aggregation in EM are discussed in detail in Section 4.3 and Section 4.4.

### 4.1. Data Description

**Figure 5:**
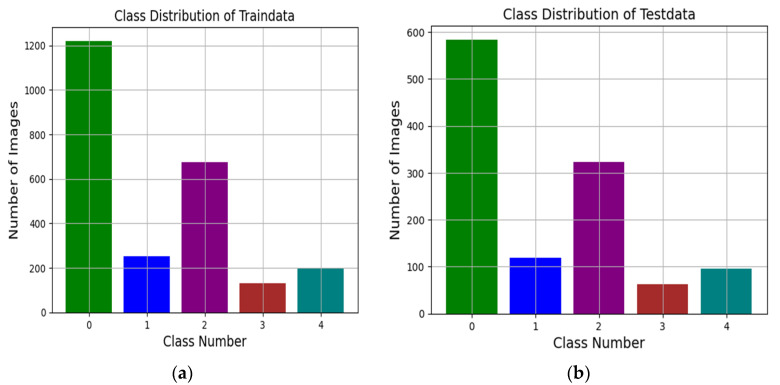
This figure depicts the class distribution of training and test data. (**a**) represents the figure of training data clearly showing uneven numbers in each class. Likewise, (**b**) represents the test data distribution that portrays the imbalanced data of classes.

The main objective of this research is to show that quantitative examination of the imaging information can give more and better information comparable to that of a physician. The input images are taken from the Kaggle dataset of the Asia Pacific Tele-Ophthalmology Society (APTOS). This dataset was first published in the third quarter of 2019 by the APTOS team and is open for competition to train and test new models. The dataset is compiled into the train (3662) and test (1928) sets, comprised of 5590 images in total and having five classes, namely 0, 1, 2, 3 and 4. To train the EM, the training dataset is further divided into trdata and tsdata with a ratio of (68%/32%) based on the trial-and-error method. As a result, the trdata set consists of 2479 FI images, while the remaining 1183 images are from the tsdata set. The trdata and tsdata are imbalanced, consisting of five classes as depicted in Figure 5, and detailed in Table 1 and Table 2, respectively. 

### 4.2. Data Distribution

As mentioned earlier, the data are imbalanced, meaning that the number of instances in each class is unequal, as shown in Table 1 and Table 2. Table 1 depicts the *trdata* having 2479 images, while Table 2 consists of the *tsdata* having 1183 images for training the baseline models in EM.

### 4.3. Baseline Model

Before proceeding to the creation of the baseline model, we have prepared our data to pass through an initial cycle, such as pre-processing. The data consisted of high-quality images, and we resized them into patches of 512×512×3 input images. After resizing, we normalized the training set by dividing each with the standard deviation (255 in the RGB image, which denotes the maximizing value of the pixel channel) so that each image value lies on [0, 1]. EM is typically built in two stages: baseline model creation and model aggregation. As discussed earlier, diverse baseline models are created using three strategies: data, parameter, and structural diversity. This paper emphasizes data diversity and elaborates in the coming section.

#### Data Diversity

The data diversity is created in the *trdata* to manipulate the input images in a disjoint or repeated way. As depicted in Figure 4, three baseline models, namely CNN-1, CNN-2, and CNN-3, are trained with the diverse dataset. CNN-1 is trained on the original *trdata* (imbalanced data) and can be seen in Table 3. Next, Class 1 and Class 2 in the original data (imbalance data) are augmented via the augmentation technique so that the number of instances in these classes is equal to those in Class 0. The augmentation techniques include position augmentations (such as scaling, rotation, and cropping) and color augmentations (such as brightness, contrast, and saturation). The augmented data increases the number of instances in each class of the *trdata* (as shown in Table 4). CNN-2 is trained with these data. Similarly, another augmented dataset is created by the affine technique so that the number of images in each class becomes equal (as shown in Table 5), resulting in balancing each class. CNN-3 is trained on this balance data. The input images or data are now varied and can be verified from Table 3, Table 4 and Table 5 and can be depicted in Figure 4, eventually heading in the direction of data diversity. The architectures and hyperparameters of the three baseline models of data diversity remain the same, as depicted in Table 6. 

### 4.4. Ensembling Using Majority Voting

The output obtained from all three baseline models discussed in Section 4.3 is aggregated using the majority vote. This method of classification is similar to averaging votes in regression problems. The predicted classes from the baseline models are counted, and the final result is evaluated based on the class with the most votes.

### 4.5. Model Evaluation

The performance of the EM on the testing dataset is evaluated using statistical metrics, including accuracy, precision, recall, F1 score, sensitivity, and specificity. These metrics rely on four key statistical characteristics of the classification model: true positives (TP), true negatives (TN), false positives (FP), and false negatives (FN). The number of correctly predicted positive and negative classes is denoted by TP and TN, respectively. Similarly, when the positive and negative classes are predicted incorrectly, they are denoted by FP and FN. A classifier’s accuracy estimates how often it is correct and can be achieved using Equation (1).

(1) $$Accuracy=\frac{TP+TN}{TP+FP+TN+FN}$$

Precision calculates the proportion of accurately predicted positives to all the positives predicted by the model. In other words, precision reflects the accuracy of favorable outcomes, as in Equation (2).

(2) $$Precision=\frac{TP}{TP+FP}$$

The proportion of true positives that the model predicts can be measured by the recall, also known as sensitivity, and is given by Equation (3).

(3) $$sensitivity/Recall=\frac{TP}{TP+FN}$$

The fraction of true negatives correctly anticipated is determined by the specificity using Equation (4).

(4) $$Specificity=\frac{TN}{TN+FP}$$

## 5. Results

**Figure 6:**
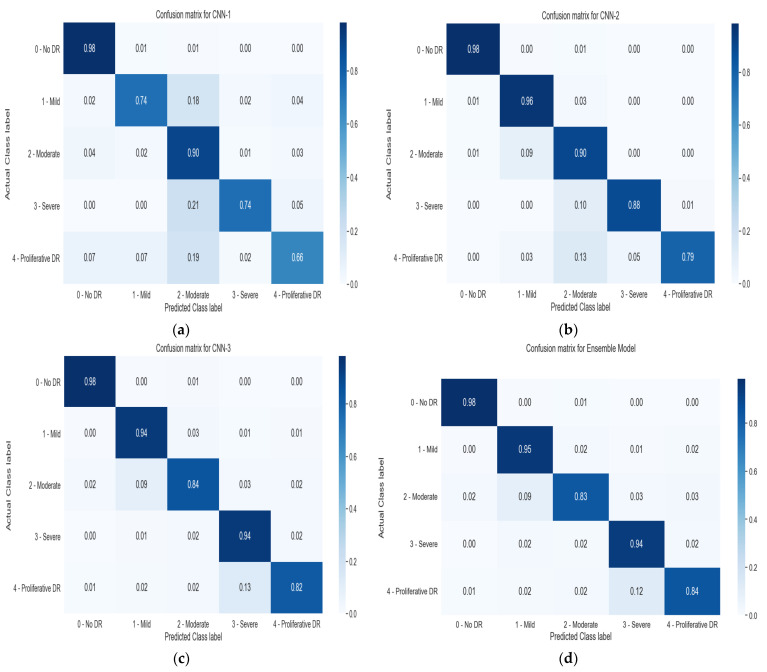
Result evaluation in the shape of a confusion matrix. (**a**) CM evaluates the result of the CNN-1 model (**b**) Achievement of a significant result for Class 1 and Class 3 (**c**) the discrepancies remaining in CNN-2 are counterbalanced in the CNN-3 model (**d**) Improved outcomes in Class 4 remain declining in the prior model.

The testing dataset is evaluated with these three baseline models as a single model and in a proposed EM for the DR dataset. Results based on the confusion matrix (CM) are shown in Figure 6. It is comprised of four subfigures Figure 6a–d, respectively. Figure 6a CM evaluates the result of the CNN-1 model, which is not good for Class 1 and Class 3. The main reason is that the data are limited and imbalanced. Figure 6b depicts that the model outperforms and achieves a significant result for Class 1 and Class 3. Reviewing the previous model, the CNN-2 model is trained to balance Class 1 and Class 2 as compared to Class 0. This way, the CNN-2 model predicts a better result than the CNN-1 model. Figure 6c shows that the discrepancies remaining in CNN-2 are counterbalanced in the CNN-3 model, and improved outcomes in Class 4 remain declining in the prior model, as can be seen in Figure 6d. 

The previous studies showed that EM compensates for each other’s error, likewise assisting the weak classifier with the strong classifier. In our case, the EM does the same; from all the prior models, CNN-based EM outer performed the result specifically for Class 1 and Class 4. Class 1 has a significant role in predicting the DR in time and can prevent patients from developing severe conditions for eyes such as retinal detachment, glaucoma, macular edema, severe and proliferative DR, etc. 

The architectures of our research work, i.e., three individual and one ensemble model, are executed on a Python software package specific to a high-end GPU. GPUs consist of 1080 CUDA cores with the NVIDIA CUDA deep neural network library (CUDNN) for GPU learning. The DL package Keras4 was implemented with the help of a ML backend library such as TensorFlow 4.0. Different hyper-parameters are used for all the tasks, such as batch size (64,128,256), optimizers (Adam), and the cross-entropy (SoftMax) loss function, to implement the code efficiently. The tabular chart results of the single CNN models and CNN-based EM are reported in Table 7 for better understanding. In addition, the proposed CNN-based EM is also compared to the models in the literature (Table 8) that employed certain DR datasets. The existing approaches are based on end-to-end learning and require a substantial amount of labeled data for training. In comparison to the existing state-of-the-art models, the presented method achieves excellent performance with an imbalanced and small number of training images, as can be seen in Table 8. 

## 6. Conclusions and Future Direction

AI and its implementations have become one of the most researched topics. In recent years, we have witnessed the progress and implementation of AI in almost every medical field. Several AI-based tools have been developed to automate MIA and improve automatic image conception. The primary objective of this automation is to help doctors and other medical professionals detect various ailments. An ADS is constructed while dealing with ML. ML is weak at learning the model automatically, while DL requires a substantial amount of data. Furthermore, ethical requirements and privacy constraints have worsened ADS construction. A CNN-based EM integrating three baseline models and automatic DR detection is achieved by categorizing images into five classes to overwhelm this shortcoming. The CNN-based EM is powerful enough to extract valuable features and automatically distinguish between the five classes. The DR classification achieved an accuracy of 91.06%, a precision of 91.00%, a sensitivity of 95.01%, and a specificity of 98.38%, even when using a limited and unbalanced dataset. Likewise, model training is performed in a DL-based EM by labeling the small and unbalanced images from the training data. Moreover, this approach relies less on medical experts and mitigates the tedious task of annotating all images.

Data diversity in a CNN-based EM could improve ensemble model performance by combining the predictions of multiple models trained on different subsets of data. 

On the other hand, data diversity in a CNN-based EM increases the model’s complexity, as training and maintaining multiple models can be computationally expensive and increase the complexity of the overall system. The system complexity hinders the ability to interpret the predictions of an EM as they are based on the combination of multiple models. If the data used to train an ensemble model is too diverse, it can lead to overfitting, where the models perform well on the training data but poorly on unseen data.

We propose the following suggestions for possible future research development:

- The suggested methodology is only validated on a single case study and can be extended to other DR case studies to make it even more useful.
- If the proposed model is employed for a future task where the data may contain noise due to variations in image quality caused by capture sensors and lighting conditions, there are various approaches to reduce the noise and improve the algorithm’s performance. These approaches include the use of median, mean, conservative smoothing, un-sharp filters, frequency filters, and Gaussian smoothing.
- Diversity plays a key role in EM, and other baseline models can be used by employing diverse strategies. It is possible to consistently generate more baseline models and blend them into a single model to further outperform a model’s performance metrics with our suggested model.

## Figures and Tables

**Table 1:** *trdata* for training the baseline models in EM.

Total Images of *trdata*						
Class Number	0	1	2	3	4	Total
Number of Images	1221	251	676	131	200	2479

**Table 2:** *tsdata* for training the baseline models in EM.

Total Images of *tsdata*						
Class Number	0	1	2	3	4	Total
Number of Images	548	119	323	62	95	1183

**Table 3:** Data distribution for CNN-1.

Total Images of *trdata*						
Class Number	0	1	2	3	4	Total
Number of Images	1221	251	676	131	200	2479

**Table 4:** Data distribution for CNN-2.

Total Images of *trdata*						
Class Number	0	1	2	3	4	Total
Number of Images	1221	1221	1221	131	200	3994

**Table 5:** Data distribution for CNN-3.

Total Images of *trdata*						
Class Number	0	1	2	3	4	Total
Number of Images	1221	1221	1221	1221	1221	6105

**Table 6:** Hyper-parameters of CNN models.

S.no	Model	Layers	Batch Size	Learning Rate	Optimizer
01	CNN-1	5	128	1$e^{-4}$	Adam
02	CNN-2	5	128	1$e^{-4}$	Adam
03	CNN-3	5	128	1$e^{-4}$	Adam

**Table 7:** Performance evaluation of our proposed model.

Model	Accuracy	Precision	Sensitivity	Specificity
CNN-1	89.87%	90%	74.11%	98.03%
CNN-2	93.74%	93%	95.89%	98.36%
CNN-3	90.81%	91%	94%	98.15%
CNN-based EM	91.06%	91%	95.01%	98.38%

**Table 8:** Comparisons of recent work with diabetic retinopathy data.

Model	Number of Images	Accuracy	Precision	Sensitivity	Specificity
CNN-ResNet34 [18] Binary Classification (BC)	Kaggle data (35126)	85.0%	-	86.0%	-
CNN-EM (BC) [19]	Kaggle data (35126)	88.21%	-	85.57%	90.85%
CNN(AlexNet) Multiple Class Classification (MCC) [21]	Kaggle (22700) and IDRiD (516)	90.07%	-	-	-
Two deep-CNN-EM are used (MCC) [33]	Kaggle data (35126)	80.36%	-	47.7%	85.94%
Ensembling Five PT model (MCC) [7]	Kaggle data (35126)	80.8%	63.8%	51.5%	86.7%
Hybrid of TL and CNN model (MCC) [26]	APTOS-2019 Kaggle data (3662)	82.18%	-	-	-
Proposed Model (CNN-based EM)	APTOS-2019 Kaggle data (3662)	91.06%	91%	95.01%	98.38%

## Data Availability

Not applicable.
